# Microglial EPOR Contribute to Sevoflurane-induced Developmental Fine Motor Deficits Through Synaptic Pruning in Mice

**DOI:** 10.1007/s12264-024-01248-5

**Published:** 2024-06-21

**Authors:** Danyi He, Xiaotong Shi, Lirong Liang, Youyi Zhao, Sanxing Ma, Shuhui Cao, Bing Liu, Zhenzhen Gao, Xiao Zhang, Ze Fan, Fang Kuang, Hui Zhang

**Affiliations:** 1 State Key Laboratory of Oral and Maxillofacial Reconstruction and Regeneration, National Clinical Research Center for Oral Diseases, Shaanxi Engineering Research Center for Dental Materials and Advanced Manufacture, Department of Anesthesiology, School of Stomatology, Fourth Military Medical University, Xi’an, 710032 China; 2 Department of Neurobiology and Institute of Neurosciences, School of Basic Medicine, Fourth Military Medical University, Xi’an, 710032 China

**Keywords:** Erythropoietin, Microglia, Synaptic pruning, Sevoflurane, Fine motor deficits

## Abstract

Clinical researches including the Mayo Anesthesia Safety in Kids (MASK) study have found that children undergoing multiple anesthesia may have a higher risk of fine motor control difficulties. However, the underlying mechanisms remain elusive. Here, we report that erythropoietin receptor (EPOR), a microglial receptor associated with phagocytic activity, was significantly downregulated in the medial prefrontal cortex of young mice after multiple sevoflurane anesthesia exposure. Importantly, we found that the inhibited erythropoietin (EPO)/EPOR signaling axis led to microglial polarization, excessive excitatory synaptic pruning, and abnormal fine motor control skills in mice with multiple anesthesia exposure, and those above-mentioned situations were fully reversed by supplementing EPO-derived peptide ARA290 by intraperitoneal injection. Together, the microglial EPOR was identified as a key mediator regulating early synaptic development in this study, which impacted sevoflurane-induced fine motor dysfunction. Moreover, ARA290 might serve as a new treatment against neurotoxicity induced by general anesthesia in clinical practice by targeting the EPO/EPOR signaling pathway.

**Supplementary Information:**

The online version contains supplementary material available at 10.1007/s12264-024-01248-5.

## Introduction

Advances in modern general anesthesia (GA) have guaranteed the successful implementation of millions of surgeries every year [1]. However, concerns have been raised about whether general anesthetic agents cause deleterious effects on the central nervous system (CNS). Animal studies have identified Alzheimer-like disease processes [2], cognitive dysfunction [3], autism [4], learning deficits [5]. and fine motor abnormalities [6, 7] in the brain receiving repeated general anesthesia. A secondary analysis based on the MASK study also concluded that exposure to multiple procedures with general anesthesia was associated with decreases in fine motor skills in subjects [8]. Subsequently, a number of studies from rodents to humans including non-human primates were motivated to focus on the issue of anesthesia-induced fine motor deficits [7, 9].

Sevoflurane is a commonly used anesthetic in pediatrics, which has been implicated in causing neurotoxic risks in immature animals and humans [10, 11]. A repeated sevoflurane exposure (3 consecutive 2-hour sections at P6-8 in mice) model has been established to induce motor dysfunctions in rodents [7]. Diverse cellular and molecular changes causing synaptic damage have been implicated in the pathogenesis [12], in which intervention target (s) might be found to aid the development of a potential drug for the treatment of GA-induced neurotoxicity. Reportedly, neuroinflammation driven by microglia is one of the primary culprits for synaptic loss in multiple developing diseases including depression [13] and anxiety-like behavior [12]. Microglia are well-described mediators that shape synaptic plasticity through their phagocytosis properties. Prior studies have indicated that a pro-inflammatory state triggered aberrant synaptic phagocytosis of microglia in the developing brain, causing abnormalities in brain functions [14]. However, whether and how sevoflurane exposure resulted in this pathological process remains unclear. Neuropsychological and imaging studies have indicated that excitatory neurons in the medial prefrontal cortex (mPFC) are critical for the regulation of fine motor [15, 16]. Hence, exploring the molecular mechanism of anesthesia-induced loss and damage of excitatory synapses with aberrant pruning of microglia in the mPFC is an appealing concept. Brain-derived EPO is a key anti-inflammatory mediator exerting neuroprotective effects via EPOR that is expressed on microglia [17]. The EPO derivative ARA290 is a widely used drug preventing neuroinflammation in multiple diseases, such as brain and peripheral nerve injury [18, 19]. We hypothesized that EPO/EPOR system in the mPFC-mediated sevoflurane-induced fine motor deficits and supplementation of the EPO derivative ARA290 could attenuate sevoflurane-induced microglia proinflammatory activation alleviated, excessive synaptic pruning and synaptic deficiency, and improved fine motor impairments in mice.

Here, we identify that inhibition of EPO/EPOR-janus kinase 2 (JAK2)-signal transducer and activator of transcription 3 (STAT3) signaling pathway mediates microglial activation and excessive synaptic phagocytosis after repeated sevoflurane exposure, resulting in impairment in fine motor coordination in mice. We further showed that injection of the EPO derivative ARA290 significantly attenuated microglia proinflammatory activation, alleviated excessive synaptic pruning and synaptic deficiency, and significantly improved fine motor impairment in the repeated sevoflurane exposed mice. Together, our results uncover the molecular signaling that regulates repeated exposure to sevoflurane-induced fine motor dysfunction, and we speculate that ARA290 might be an efficient supplementation to sevoflurane-induced fine motor deficits in clinical conditions.

### Materials and Methods

#### Animals

Adult C57BL/6 mice were purchased from the Animal Center of the Fourth Military Medical University and raised in a standard environment with a temperature of 22–25°C, a humidity of 50%, and a 12-h/12-h light/dark cycle. 5–6 mice were reared in each cage and allowed ad libitum access to food and water. After housing male and female mice together, the pregnant females were isolated and fed till their delivery. The newborn pups (including both males and females) were used for studies. All experimental procedures were performed following the institutional guidelines and approved by the Animal Care and Use Committee of the Fourth Military Medical University.

### Mice Treatments

Newborn C57BL/6 mice were randomly divided on the 6th day after birth (P6) into the following groups. (1) Control (Ctl) group: mice inhaled 60% oxygen (balanced with nitrogen) for 2 hours every day for three days in a row. (2) Sevoflurane (Sev) group: Mice inhaled 3% sevoflurane (Cat#: H20110142, Baxter) + 60% oxygen (balanced with nitrogen) for 2 h/day for three days in a row. (3) Ctl+PBS group: mice inhaled 60% oxygen (balanced with nitrogen) for 2 hours every day and intraperitoneally injected 50 μL PBS (Cat#: PC-00003, Plant Chem Med) for three days in a row. (4) Sev+PBS group: Mice received 3% sevoflurane exposure and were treated with an intraperitoneal injection of 50 μL PBS for three days in a row. (5) Sev+ARA290 group: Mice were intraperitoneally injected with 50 μL ARA290 (30 μg/kg) (Cat#: PC-49529, Plant Chem Med) after sevoflurane anesthesia. The model mice were placed separately in a rectangular transparent sealed Plexiglas container (length × width × height = 25 cm × 12 cm × 10 cm), which was connected to an oxygen and/or sevoflurane catheter, and a warm blanket was placed at the bottom to maintain body temperature. The respiration and skin color of mice were constantly observed during the molding process. After completing the modeling, neonatal mice were quickly placed back next to the dams. All procedures were performed from 9:00 to 11:00 AM on the day.

### Golgi Staining

The abovementioned mice were anesthetized by intraperitoneal injection of pentobarbital sodium (150 mg/kg) and perfused with PBS at P60. Mouse brains were dissected and soaked in Golgi dyeing liquor containing 50% potassium dichromate (Cat#: 021563389, MP Biomedicals), 5% mercury chloride (Cat#: M1136, Sigma Aldrich), and 5% potassium chromate (Cat#: 529508, Sigma Aldrich). After 6 days of protecting light, 150 μm brain slices were prepared and rinsed with distilled water, dehydrated with ethanol, and then treated with ammonia (3:1). Next, slices were rinsed and hatched with 5% sodium thiosulfate for 10 min, followed by dehydration with graded ethanol and clarification with xylene. The completed slices were observed under the bright field of view of the Olympus FV1000. The images were captured by z-stack scanning with an excitation wavelength of 405 nm. Sholl analysis was conducted on the branching of dendrites. Imaris software (version 8.4.1, Bitplane) was applied to rebuild the dendrite axis and dendritic spine of pyramidal neurons in the mPFC.

### Transmission *Electron* Microscopy

Mice were anesthetized by intraperitoneal injection of pentobarbital sodium (150 mg/kg) and perfused with physiological saline (Cat#: 2205200721, CISEN) and then postfixed with 4% polyformaldehyde mixture including 1% glutaraldehyde. Histologic slices of 50 μm were prepared using a vibratome (VT1000 S, Leica) and then fastened with 1% osmium tetroxide, dewatered with gradient ethanol, treated with propylene oxide, and embedded in Epon 812 (SPI-Chem). Ultrathin slices (70–80 nm) were ready on an ultramicrotome (EMUC6, Leica), fixed on mesh grids (6–8 slices per grid). After redying with uranyl acetate and lead citrate, the sections were observed under JEM-1230 electron microscopy (JEOL), and synaptic images were collected.

### Western Blot

The brain tissue (mPFC) was homogenized in RIPA lysis buffer (0.5% NP-40, 150 mmol/L NaCl, 1 mmol/L EDTA, 10 mmol/L Tris, and 1% Triton X-100 at pH7.4) including protease (Cat#: 43002700, Roche) and phosphatase inhibitor (Cat#: 41659200, Roche). The concentration of protein was measured by BCA assay (Cat#: 23225, Thermo Scientific). After separation by 10% or 12% SDS-PAGE, the protein was deflected to 0.22 μm polyvinylidene fluoride membranes (Cat#: 03010040001, Roche Diagnostics Gmbh), blockaded with TBS including 5% skim milk and 0.1% Tween 20, and hatched with the primary antibodies overnight at 4°C as shown below: mouse anti-β-actin (1:5000, GeneTex#11003), rabbit anti-β-actin (1:5000, GeneTex#109639), rabbit anti-iNOS (1:1000, proteintech#18985-1-AP), rabbit anti-Arginase-1 (1:2000, proteintech#16001-1-AP), rabbit anti-IL-1β (1:1000, CST#31202s), rabbit anti-LAMP1 (1:1000, proteintech#21997-1-AP), mouse anti-CD68 (1:1500, Bio-Rad#MCA1957), rabbit anti-EPOR (1:1000, GeneTex#37704), mouse anti-EPO (1:200, Santa Cruz#5290), rabbit anti-p-JAK2 (1:500, CST#3771), rabbit anti-JAK2 (1:1000, CST#3230), rabbit anti-p-STAT3 (1:1000, CST#9145), rabbit anti-STAT3 (1:1000, GeneTex#636400), goat anti-Iba1 (1:1000, abcam#5076), rabbit anti-HIF2*α* (1:1000, abcam#109616). After washing with TBST, the membranes were hatched with HRP-conjugated anti-rabbit (1:5000, Proteintech#SA00001-2), anti-mouse (1:5000, Proteintech#SA00001-1), and anti-goat (1:5000, Proteintech#SA00001-4), respectively at room temperature for 2 hours. The bands were displayed with the ECL reagent kit (Cat#: 32106, Thermo). The grayscale value was quantified by ImageJ to determine the level of target proteins relative to β-actin.

### Immunostaining

Mice were anesthetized by intraperitoneal injection of pentobarbital sodium (150 mg/kg) and transcardially perfused with saline followed by 4% paraformaldehyde (PFA). Brains were dissected, fixed for 4 h in 4% PFA, and kept in 30% sucrose at 4 °C until the brain sank to the bottom of the container. Serial coronal 25 μm slices were prepared and blockaded by PBS including 3% BSA and 0.3% Triton-X100 and then hatched with primary antibodies overnight at 4 °C as shown below: rabbit anti-Iba1 (1:800, Wako Chemicals#019-19741), guinea-pig anti-VGlut2 (1:200, Synaptic Systems#135404), mouse anti-CD68 (1:500, Biolegend#137001). After washing with PBS, the reciprocal secondary antibodies conjugated with Alexa Fluro 488 (donkey anti-rabbit) or Alexa Fluro 594 (donkey anti-mouse) were incubated with the slices at room temperature in the dark for 2 h. After washing with PBS, the slices were redyed with Hoechst (1:1000, Sigma#33324) for 15 min. Fluorescent images were acquired using a confocal microscope (Olympus Fluoview Ver4.2 b) with a 20×, 40× or 100× oil objective. All images were taken from the mPFC.

### Dual Fluorescence *in situ* Hybridization and Immunofluorescence Staining

Fluorescent in situ hybridization was conducted using the RNAscope Multiplex Fluorescent V2 Assay kit (Cat#: 323100, ACD) in line with the manufacturer’s instructions on the brain slices. EPOR (Cat#: 412351, ACD) probes were applied in the current research. In situ hybridization was followed by immunofluorescence staining, as described above, by using rabbit anti-Iba1 (Cat#: 019-19741, Wako Chemicals), rabbit anti-NeuN (1:800, Millipore#ABN78) and rabbit anti-S100β (1:100, abcam#ab52642). Alexa Fluor 488 Goat anti-rabbit IgG (Cat#: 706585148, Jackson immunoresearch) secondary antibody was applied in dual fluorescence in situ and immunofluorescence staining.

### 3D Reconstruction of Images and Synapse Engulfment Quantification

Z-stacks encompassing high-magnification images were collected on a Zeiss laser scanning confocal microscope with a 100x objective using 0.5 µm z steps. Laser power and gain were consistent across all experiments. For each hemisphere, at least 8 fields were imaged. Subsequently, all channels of images were subjected to background subtraction and Gaussian filtering using IMARIS 9.3.1 software (Bitplane). Then, 3D volume surface renderings were created. For the analysis of microglia-synapse engulfment, a threshold was selected to include as many of the processes as possible while excluding any background, and a 0.01 µm^3^ size filter was applied. The automatic threshold calculated in Imaris based on k-means statistical methods was used in the majority of analyses. Then, presynaptic VGlut2^+^ boutons were reconstructed as ‘‘spots’’ of 0.8 mm diameter (corresponding to the largest measured size of presynaptic boutons) and their total number was automatically calculated. Next, the number of spots located at no more than 0.5 mm from the microglia surface was automatically determined and indicated as a contact. The 0.5 mm distance was calculated from the center of mass of the reconstructed ‘‘spot’’. It corresponded to its radius thus identifying as contact only those spots that were completely juxtaposed to the surface. In the representative images only contacted boutons were shown as reconstructed spots.

### RNA-Seq

Brain tissues (mPFC) from the Ctl and Sev groups on the third day after repeated sevoflurane or oxygen exposure were harvested (*n = *3 per group) under RNase-free conditions. Messenger RNA (mRNA) sequencing was conducted by Novogene Co, Ltd (Xi’an, China). Briefly, general RNAs were separated from the mPFC making use of TRIzol Reagent (Cat#: 15596018, Ambion) and then used to build a sequencing library. mRNA was enriched and cleaved into short fragments making use of fragment buffer, followed by reverse transcription into cDNA using random primers. The cDNA fragments were purified, performed end repair, poly (A) tails, and then linked to the Illumina sequencing adapter. Then the linked product was selected by agarose gel electrophoresis, amplified by PCR, and sequenced by Illumina Novaseq6000 (Denovo biotechnology). Differentially expressed genes (DEGs) were evaluated by analyzing differential RNA expressions between two groups. Transcripts with false discovery rate (FDR) parameters below 0.05 and an absolute fold change of 2 or greater were considered differentially expressed. Pathway enrichment analysis was conducted using the Kyoto Encyclopedia of Genes and Genomes (KEGG). The calculated P-value was corrected by FDR, with an FDR of 0.05 or less as the threshold. The pathway that meets this condition was defined as the significantly enriched pathway in DEG.

### Quantitative Real-Time PCR

General RNAs from the mPFC were harvested making use of TRIzol (Cat#: 15596018, Ambion) in the light of the manufacturer’s instructions, followed by reverse transcription into cDNA making use of the Prime Script RT Reagent Kit (Cat#: RR036A, TaKaRa). The standard SYBR Green (Cat#: B21202, Bimake) method was applied to test the relative expression of mRNA using a StepOnePlus Real-Time PCR System (Applied Biosystems). The expression of the object gene was standardized to the level of GAPDH. Primers and probes used are listed in Supplemental Table S1.

### ELISA

Serum and viscus EPO levels were detected using a commercially available mouse erythropoietin (EPO) ELISA kit (Cat#: F10430, Shanghai Xitang Biotechnology Co.). Briefly, the diluted and standard specimens were added to the well and hatched at 37°C for 90 min. Then, the plate was washed, tested antibodies, and hatched at 37°C for 1 h. The plate was washed again, Avidin-Biotin Peroxidase Complex in every well, and then hatched at 37°C for 30 min. The plate was washed and added color developing reagent. After hatching at 37°C for 15–25 min, the plate was added to the stopping solution and read the optical density at a wavelength of 450 nm. All experiments were duplicated three times. The results of EPO were expressed in concentration as pg/mL.

### Gait Analysis

Automated gait assessment tests are used in studies of disorders characterized by gait impairment including fine motor dysfunction [20]. The CatWalkXT^®^ (Version 10.6, Nordus) of the automatic gait analysis system (Nordus Information Technology) is composed of a walkway covered by a tunnel (a 1.3-meter horizontal glass panel). Briefly, the animals walked down a glass aisle while the camera was recording the footprints from beneath. After presupposing camera gain (17.0 dB) and intensity threshold (0.1), no less than 30 runs were recorded for every animal to gain representative runs completed by every animal. When animals walk continuously without stopping on the walkway, a single run is completed. The data captured by CatWalkXT^®^ were semi-automatically analyzed by software. Data on indicators such as paw print area, paw force, and supported paw base were collected. After initial adaptation to the new environment, animals must perform at least three uninterrupted runs to meet the conditions for gait analysis.

### Rotarod Test

An accelerating rotarod apparatus was utilized to detect the motor coordination ability of mice. Firstly, mice were trained with 3 trials daily for 3 days. On the first two days, the accelerating speeds were 0-5 r/min and 0–10 r/min within 10 s, respectively. The speed was constantly maintained for 5 min. On the third day, the speed was accelerated from 0-5 r/min within 10 s, then accelerated to 20 r/min within 5 min. The detection stage was on the fourth day. Within 10 s, the speed was accelerated from 0–5 r/min, then accelerated to 40 r/min in 90 s. The speed was constantly maintained for 5 min. If the mice fell in the first three days, they should be placed back on the stick. On the last day, the fall time was recorded and taken as the average.

### Statistical Analysis

Data analysis was conducted with GraphPad Prism (Version 8.0) and SPSS software (Version 21.0). The test of normality was conducted with the Shapiro-Wilk test. The homogeneity of variance test was conducted with Levene's test. The data that satisfied these two requirements were analyzed by applying a two-tailed, unpaired, or paired *t*-test, a one-way ANOVA, or a repeated-measures ANOVA followed by Tukey's multiple-comparison test. The data set with non-normal distribution was analyzed by a non-parametric test. The data were denoted as the mean ± SD. *P* value less than 0.05 was considered to be statistically significant. Using PASS software (NCSS, LLC) and the results of previous studies [21], the estimated sample size (90% power and α < 0.05 [2-tailed *t*-test]) is as follows: behavioral test 12 mice per group, EPO ELISA test 12 mice per group, immunostaining 6 mice per group and western blot 3 mice per group. To reduce bias, mice with similar general conditions were randomly assigned to different groups. All experiments and data analyses were strictly conducted to control for information bias. mRNA sequencing and analysis were conducted by blinded investigators.

## Results

### Repeated Sevoflurane Exposure in Newborn Mice Leads to Long-Lasting Fine Motor Deficits

**Fig. 1:**
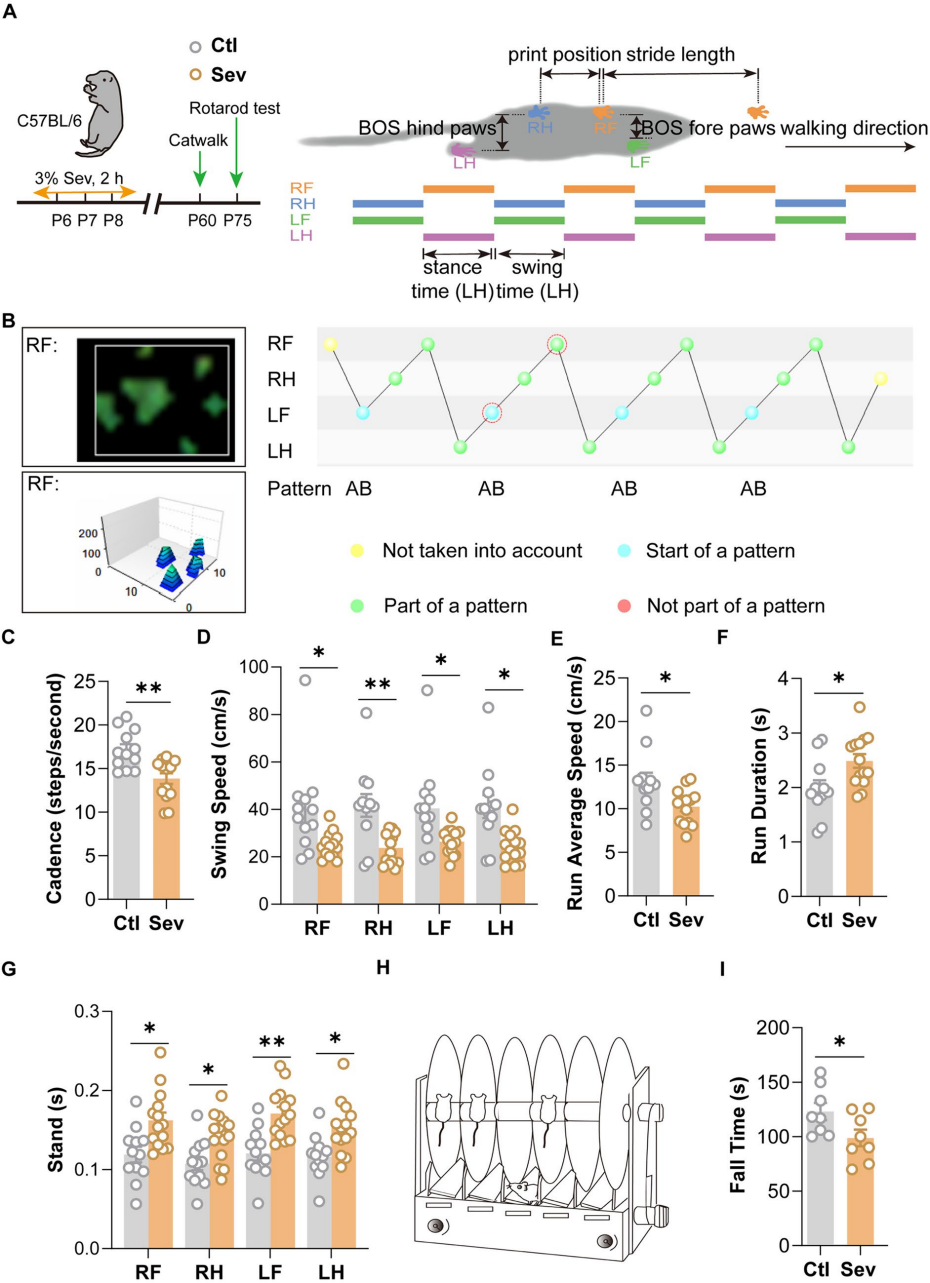
Repeated exposure to sevoflurane in the neonatal stage has a remarkable impact on fine motor function in mice. **A**, **B** Schematic diagram of the procedure for gait analysis. **C**–**G** Gaits analysis of the Ctl (*n = *12) and Sev group (*n = *14). The parameters include cadence (**C**), swing speed (**D**), run average speed (**E**), run duration (**F**), and stand duration (**G**). **H** Schematic diagram of rotarod test. **I** Latency of fall down of the rotarod test for the Ctl (*n = *8) and Sev group (*n = *8). The data are denoted as the mean ± SD. *P < 0.05, **P < 0.01, ***P < 0.001, ****P < 0.0001 (unpaired separate variance estimation *t* test).

Gait analysis is an objective tool for evaluating fine motor function in mice [22]. We conducted a gait analysis to evaluate the fine motor function of the mice at P60 (Fig. 1A, B). Results showed that repeated sevoflurane exposure caused a remarkable decrease in walking speed, including cadence, swing speed, and run average speed (Fig. 1C–E). Accordingly, extension in completion and standing time was significantly increased in the Sev group (Fig. 1F–G). There was no remarkable difference in other static indicators, including the base of support-front/hind paws, duty cycle, max contact area, mean intensity, and run maximum variation (Fig. S1A–F). Besides, the rotarod test showed a noticeable decrease in the descending latency in the Sev group (Fig. 1H–I). These results reveal that repeated sevoflurane exposure in newborn mice leads to defects in fine motor in adulthood.

### Repeated Sevoflurane Exposure Triggers Microglia Activation and Enhanced Phagocytosis in the mPFC of the Newborn Mice

**Fig. 2:**
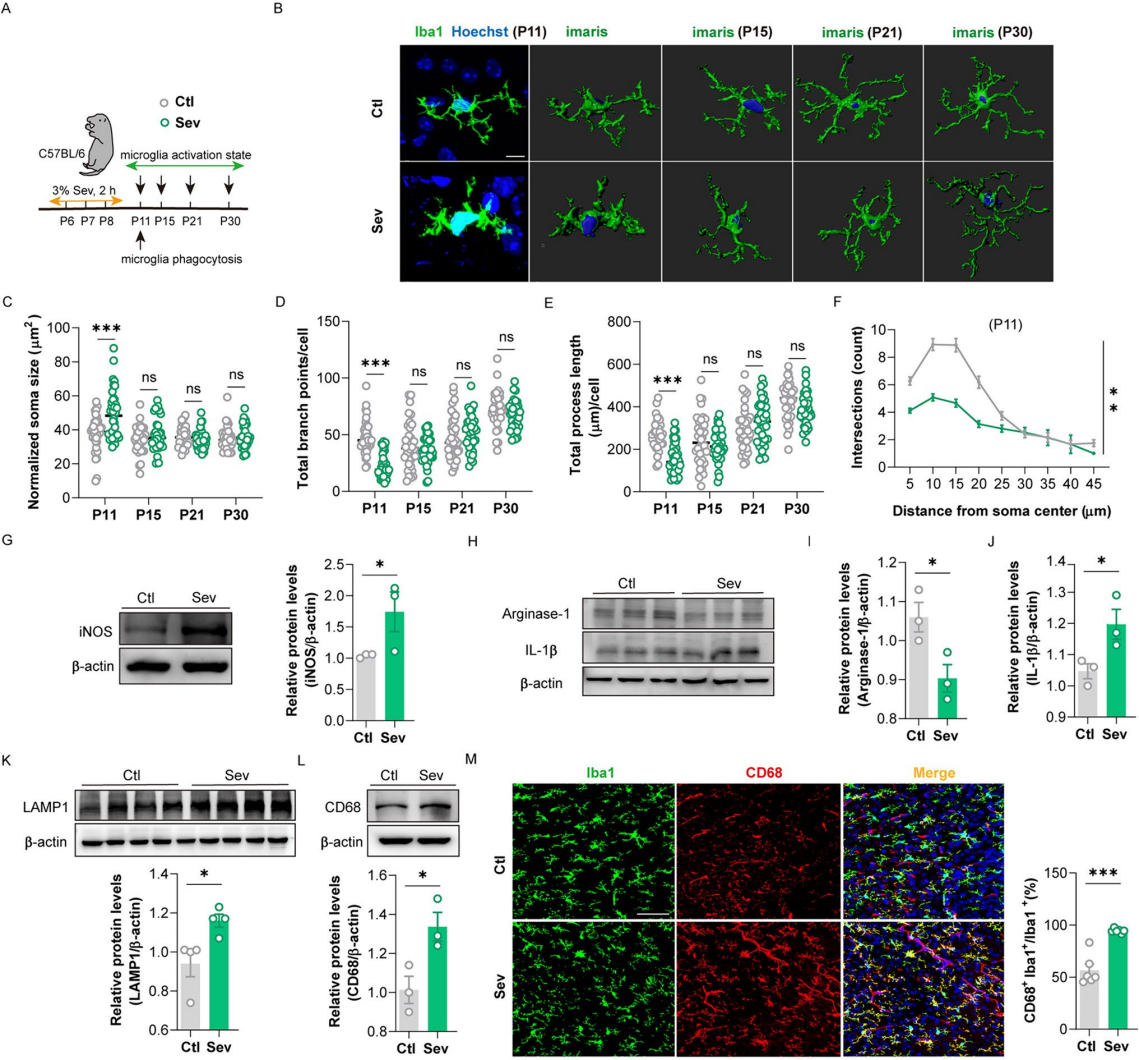
Repeated exposure to sevoflurane in neonatal mice significantly affects the status of microglia and enhances their phagocytosis. **A** Schematic diagram of observing time points. **B** The representative three-dimensional (3D) images of microglia labeled with Iba1 in the Ctl and Sev group were reconstructed with Imaris software on the 3rd (P11), 7th (P15), 13th (P21), and 22nd (P30) days after repeated sevoflurane exposure (scale bar, 10 µm), *n = *6, 36 cells. *Blue* represents the nucleus, *green* represents the branch. **C**–**E**. Quantification of the soma size (**C**), the branch points (**D**), and process length (**E**) (unpaired separate variance estimation *t*-test). **F** Sholl analysis of microglia morphology (Friedman’s M-test). **G** The protein level and quantification of iNOS in the mPFC on the 3rd day after repeated exposure to sevoflurane (unpaired separate variance estimation *t*-test), *n = *3. **H** The protein levels of arginase-1 and IL-1β in the mPFC on the 3rd day after repeated exposure to sevoflurane, *n = *3. **I**–**J** Quantification of arginase-1 (**I**) and IL-1β (**J**) protein level (unpaired separate variance estimation *t*-test). **K** The protein level and quantification of homologous lysosomal marker LAMP1 in the mPFC on the 3rd day after repeated exposure to sevoflurane (unpaired separate variance estimation *t*-test), *n = *4. **L** The protein level and quantification of lysosomal marker CD68 of microglia in the mPFC on the 3rd day after repeated exposure to sevoflurane (unpaired separate variance estimation *t*-test), *n = *3. **M** Representative images of Iba1 positive cells with CD68 immunolabeling in the mPFC on the 3rd day after repeated sevoflurane exposure (scale bar, 50 µm) and quantification of microglia engulfment capacity (CD68 immunoreactivity per cell) (unpaired separate variance estimation *t*-test), *n = *6. The data are denoted as the mean ± SD. *P < 0.05, **P < 0.01, ***P < 0.001, ****P < 0.0001.

Microglia, as immune macrophages in the CNS, are prone to morphological changes and transition to an activated state after receiving external stimuli [23]. Morphological changes of microglia in the mPFC were evaluated after repeated sevoflurane exposure (Fig. 2A). We first observed that the expression and fluorescence intensity of Iba1 was not changed in the Sev group (Fig. S1A–D). Then, the stack scanning images of microglia were rebuilt in three dimensions by applying Imaris software (Fig. 2B). We found that on the 3rd day after repeated sevoflurane exposure, the morphology of microglia in the mPFC remarkably changed, including significantly increased soma size (Fig. 2C), decreased branch points (Fig. 2D), reduced process length (Fig. 2E) and declined complexity of branches (Fig. 2F). There were no significant differences in the morphology of microglia at other timepoints (Fig. S1E-G). WB analysis displayed that the expressions of iNOS (M1 markers of microglia activation) (Fig. 2G) and pro-inflammatory factor IL-1β (Fig. 2H–J) in the mPFC were significantly increased, yet the expression of arginase-1 (M2 marker of microglia) (Fig. 2H–I) was downregulated on the 3rd day. Similarly, the mRNA level of the pro-inflammatory factor (IL-1β) was higher (Fig. S1I), while the mRNA level of the anti-inflammatory mediator (arginase-1) was decreased (Fig. S1H). We further observed the phagocytosis of microglia. We assessed whether protein levels of lysosome markers were affected in mice (since phagocytosed particles are ultimately passed to lysosomes for digestion). WB analysis and immunofluorescence displayed that the level of homologous lysosomal marker LAMP1 and microglia lysosomal marker CD68 were significantly increased (Fig. 2K–M), suggesting that repeated sevoflurane exposure in newborn mice enhanced the phagocytic activity of microglia. Together, these data demonstrate that microglia in the mPFC are activated into a pro-inflammatory state and exhibit enhanced phagocytic activity on the 3rd day after repeated sevoflurane exposure.

### Repeated Sevoflurane Exposure Caused Hyper-Phagocytosis of Excitatory Synapses in Microglia and Long-Term Synaptic Toxicity

**Fig. 3:**
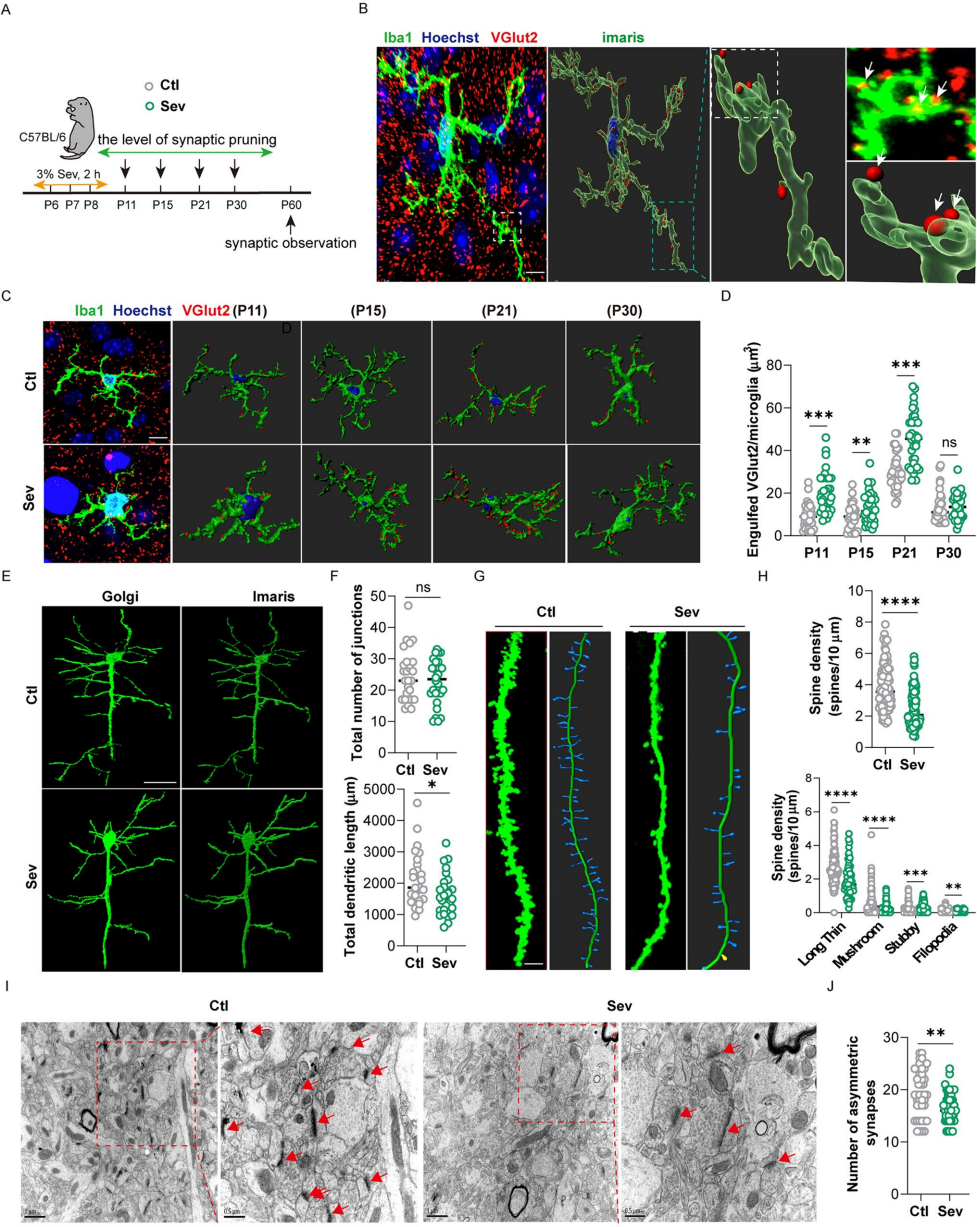
Repeated exposure to sevoflurane in neonatal mice increases the phagocytosis of microglia on excitatory synapses and long-term synaptic toxicity. **A** Schematic diagram of observing time points. **B** Images and 3D reconstruction of microglia processes (*Iba1*^+^) contacting VGlut2 **C** The representative 3D reconstruction images of microglia engulfing excitatory synapses at various time points (scale bar, 10 µm), *n = *6, 36 cells. **D** Quantification of phagocytosis of excitatory synapse by microglia. **E**. Representative images and 3D reconstruction of Golgi-stained pyramidal neurons in the mPFC of the Ctl and Sev group (scale bar, 25 µm), *n = *6, 24–28 pyramidal neurons. **F** Sholl analysis of the morphology of pyramidal neurons. **G** Representative images and 3D reconstruction of Golgi-stained dendrites of pyramidal neurons in the mPFC of the Ctl and Sev group (scale bar, 10 µm), *n = *6, 84–90 dendrites. **H** Quantitative analysis of the density of all spines and four different types of spines based on Golgi staining. **I** Representative images of TEM in the mPFC of the Ctl and Sev group (arrows, excitatory synapse; scale bars, 1 μm [left] and 0.5 μm [right]). **J** Quantitative analysis of excitatory synapses, *n = *6. The data are denoted as the mean ± SD. *P < 0.05, **P < 0.01, ***P < 0.001, ****P < 0.0001 (unpaired separate variance estimation *t* test).

Aberrant microglial phagocytosis could result in impaired pruning of synapses during development [24]. Here, we observed microglial pruning of synapses in the mPFC of the mice (Fig. 3A). We co-labeled the excitatory synapses *VGlut2*^+^ with Iba1 and found that microglia in the mPFC pruned significantly more excitatory synapses on the 3rd (P11), 7th (P15), 13th (P21) days after sevoflurane exposure (Fig. 3B, C). The most obvious pruning occurred at P21, but there was no remarkable difference at P30 (Fig. 3D). To verify the effect of microglia synaptic pruning on synaptic development, we observed dendritic spines and synapses in the mPFC in adulthood. Golgi staining showed that the number of junctions was not significantly diminished (Fig. 3E, F), but the total length of the dendrites of pyramidal neurons was significantly decreased (Fig. 3F), and the spinal density was dramatically lowered in the Sev group (Fig. 3G, H). The number of four kinds of dendritic spines was significantly reduced (Fig. 3H). With TEM, we found the density of excitatory synapses was also dramatically decreased (Fig. 3I, J). These data illustrate that repeated sevoflurane exposure in newborn mice causes over-pruning of excitatory synapses and long-lasting synaptic toxicity in the mPFC.

### EPO/EPOR-JAK2-STAT3 Signaling was Inhibited after Repeated Sevoflurane Exposure

**Fig. 4:**
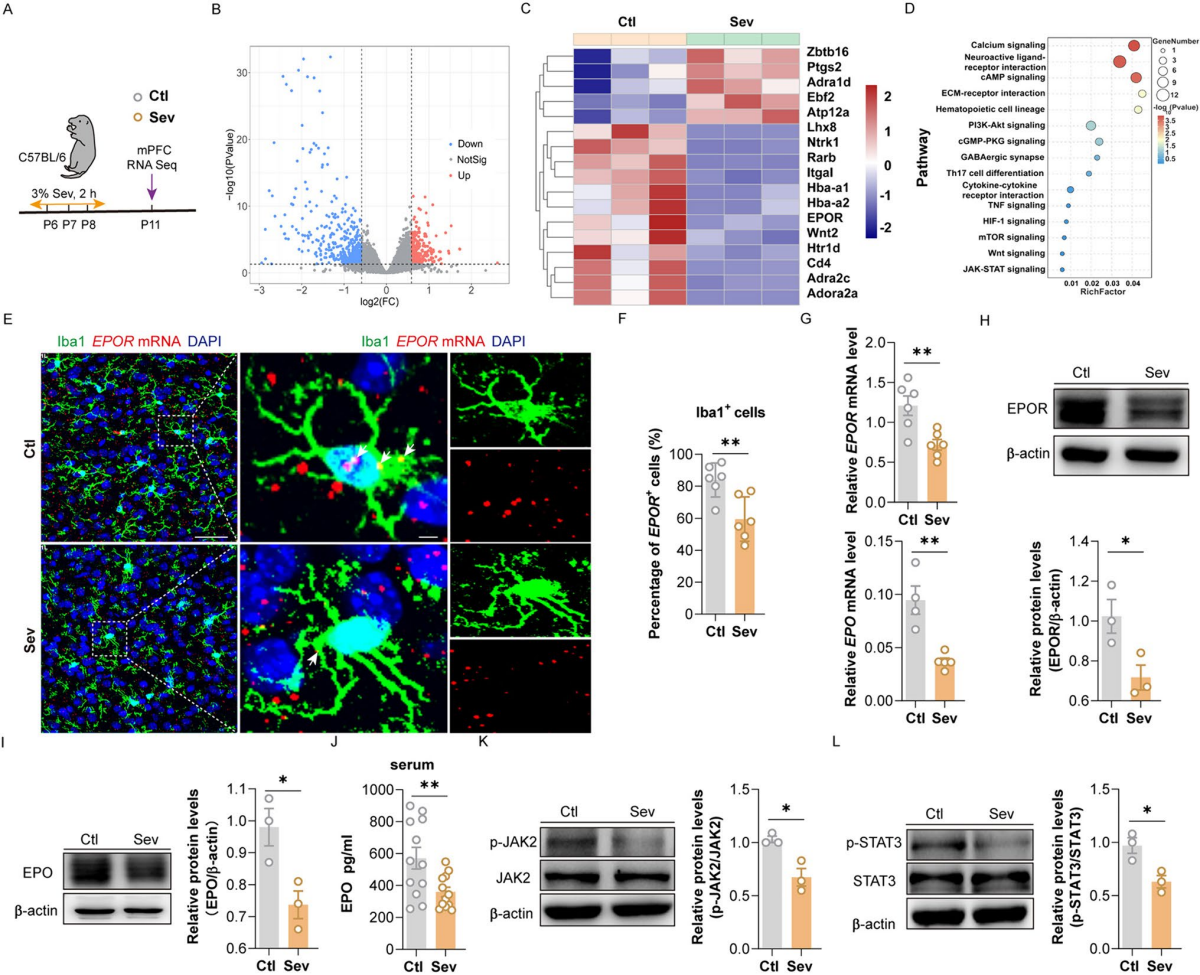
Repeated exposure to sevoflurane inhibits the EPO/EPOR-JAK2-STAT3 signal pathway. **A** Schematic diagram of time points for sevoflurane exposure and RNA-Seq. **B** Volcano plot showing RNA-Seq data from the Ctl and Sev groups. DEGs are designated in *red* (upregulation [up]) and *blue* (downregulation [down]) and are defined as having an FDR of less than 0.05. FC, fold change. **C** Comparative expression levels are shown for genes in the Sev group by comparison with the Ctl group, *n = *3. **D** The bubble plots showing KEGG pathway analysis of DEGs in the Sev group by comparison with the Ctl group. **E** RNAscope in situ hybridization and immunohistochemistry staining showing colocalization of Iba1 and *EPOR* mRNA in the mPFC of the Ctl and Sev group on the 3rd day after repeated exposure to sevoflurane, scale bars, left: 25 mm, middle and right: 10 mm. **F** Quantification of the percentage of *EPOR*^+^ cells in Iba1^+^ cells, *n = *6. **G** The mRNA (*n = *4–6) level of EPOR and EPO in the mPFC of the Ctl and Sev group on the 3rd day after repeated exposure to sevoflurane. **H**–**I** The protein level of EPOR (**H**) and EPO (**I**) in the mPFC of the Ctl and Sev group on the 3rd day after repeated exposure to sevoflurane, *n = *3. **J** The expression level of EPO in the serum of two groups on the 3rd day after repeated exposure to sevoflurane, *n = *12. **K**–**L** The protein level of p-JAK2 (**K**) and p-STAT3 (**L**) in the mPFC of the Ctl and Sev group on the 3rd day after repeated exposure to sevoflurane, *n = *3. The data are denoted as the mean ± SD. *P < 0.05, **P < 0.01, ***P < 0.001, ****P < 0.0001 (unpaired separate variance estimation t test).

We analyzed the transcriptome in the mPFC to determine the intracellular signaling involved in fine motor deficits caused by repeated sevoflurane exposure (Fig. 4A). A total of 197 DEGs (differentially expressed genes) were detected, of which 177 were downregulated and 20 were upregulated (Fig. 4B). Neuroinflammation is the most commonly mentioned mechanism and one of the primary culprits when talking about anesthesia-induced neurodevelopmental impairment [25]. EPOR, a hemoglobin-related gene related to anti-neuroinflammation in the CNS, was identified to be a downregulated gene (Fig. 4C). KEGG (kyoto encyclopedia of Genes and Genomes) analysis showeS1d that EPOR was mainly enriched in the JAK-STAT signal pathway (Fig. 4D). We tested the EPOR expression in microglia (Fig. 4E), neurons (Fig. A) and astrocytes (Fig. S1C) by performing RNAscope in situ hybridization in the mPFC of the Ctl and Sev groups with dual-color staining of neurocyte markers (Iba1, NeuN and S100β) and *EPOR* mRNA on the 3rd day after repeated exposure to sevoflurane. To determine the degree of EPOR expression in nerve cells after sevoflurane exposure, we calculated the percentage of double-labeled in microglia, neurons, and astrocytes. We found that EPOR was mainly expressed on microglia, followed by neurons, and almost not on astrocytes. After sevoflurane exposure, the microglial EPOR was significantly reduced (Fig. 4F), while the other two did not show significant changes (Fig. S1A-S3D). By using qPCR (quantitative polymerase chain reaction) and WB analyses, we proved that the mRNA and protein levels of EPOR and EPO in the mPFC were lower in the Sev group (Fig. 4G–I). Then, we confirmed the level of peripheral EPO after repeated sevoflurane exposure. According to ELISA analysis, EPO level in the serum was lower in the Sev group (Fig. 4J), while there was no remarkable difference in visceral EPO between the two groups (Fig. S1A). The protein levels of p-JAK2 and p-STAT3 in the mPFC declined (Fig. 4K, L). These data indicate that repeated sevoflurane exposure during the neonatal period inhibits the EPO/EPOR-JAK2-STAT3 signal pathway in the mPFC.

### Supplementation of ARA290 Suppresses Microglia Activation and Excessive Pruning of Developmental Synapses

**Fig. 5:**
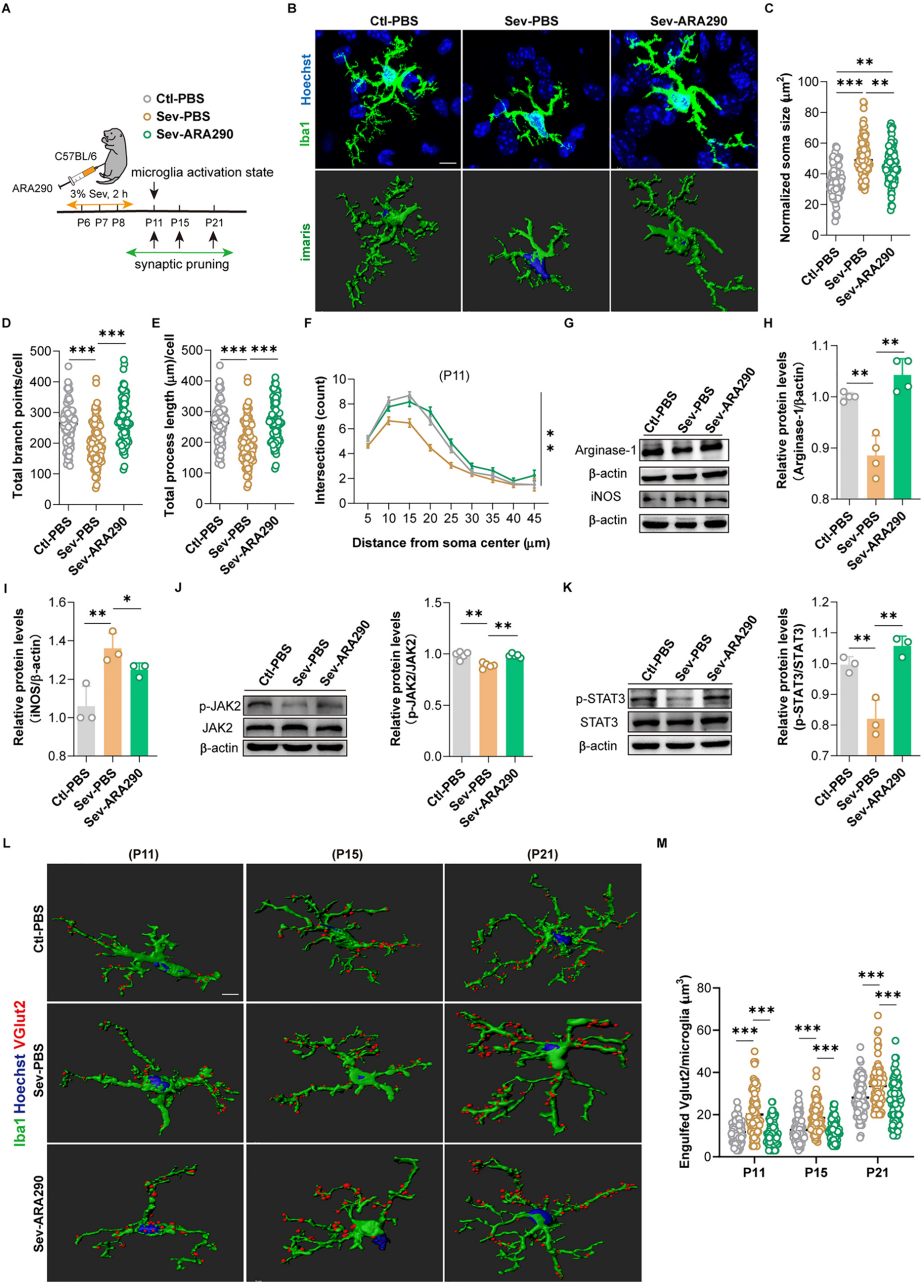
Effects of ARA290 supplementation on microglia state and synaptic pruning. **A** Schematic diagram of ARA290 administration and observing time points. **B** Representative 3D images of microglia labeled with Iba1 and reconstructed using Imaris software (scale bar, 10 µm), *n = *6, 48 cells. *Blue* represents the nucleus, and *green* represents the branch. **C–E** Quantification of the soma size (**C**), branch points (**D**), and process length (**E**) (Kruskal–Wallis H test with Nemenyi’s multiple-comparison test). **F** Sholl analysis of microglia morphology (Friedman’s M-test). **G** The protein levels of iNOS and arginase-1 in the mPFC after ARA290 supplementation, *n = *3. **H**–**I** Quantification of arginase-1 (**H**) and iNOS (**I**) protein levels (Kruskal Wallis H test with Nemenyi's multiple comparison test). **J**–**K** The protein levels and quantification of p-JAK2 (**J**) and p-STAT3 (**K**) in the mPFC after ARA290 supplementation (Kruskal Wallis H test with Nemenyi's multiple comparison test), *n = *3–5. **L** The representative 3D reconstruction images of microglia engulfing excitatory synapses at various time points after ARA290 supplementation (scale bar, 10 µm), *n = *6, 36 cells. **M** Quantification of phagocytosis of the excitatory synapse by microglia (Kruskal Wallis H test with Nemenyi's multiple comparison test). The data are denoted as the mean ± SD. *P < 0.05, **P < 0.01, ***P < 0.001, ****P < 0.0001.

We wondered whether ARA290, a derivative of EPO, could inhibit microglia activation and excessive pruning of developmental synapses induced by repeated sevoflurane exposure. We administered 30 µg/kg ARA290 intraperitoneally due to the widespread use of this dose in mouse studies [26, 27] and our validation (Fig. S1E, F). After ARA290 supplementation, we found smaller soma size, more branch points, longer process length, and more complex branches compared to the Sev-PBS group (Fig. 5B–F). WB analysis displayed that ARA290 supplementation significantly elevated the protein level of arginase-1 (Fig. 5G, H), and significantly decreased iNOS (Fig. 5G–I). Meanwhile, the protein levels of p-JAK2 and p-STAT3 returned to similar levels as the Ctl-PBS group (Fig. 5J, K). The effect of ARA290 supplementation on synaptic pruning of microglia was evaluated via the co-localization of excitatory synapses *VGlut2*^+^ with Iba1 (Fig. 5L). After ARA290 supplementation, the synaptic pruning returned to a level similar to the Ctl-PBS group on the 3rd (P11), 7th (P15), and 13th (P21) days (Fig. 5M). Together, these data indicate that ARA290 supplementation inhibits microglia activation and excessive pruning of developmental synapses through the EPO/EPOR-JAK2-STAT3 signal pathway after sevoflurane exposure.

### Supplementation of ARA290 Mitigates Long-Lasting Synaptic Toxicity and Fine Motor Defects Caused by Repeated Sevoflurane Exposure

**Fig. 6:**
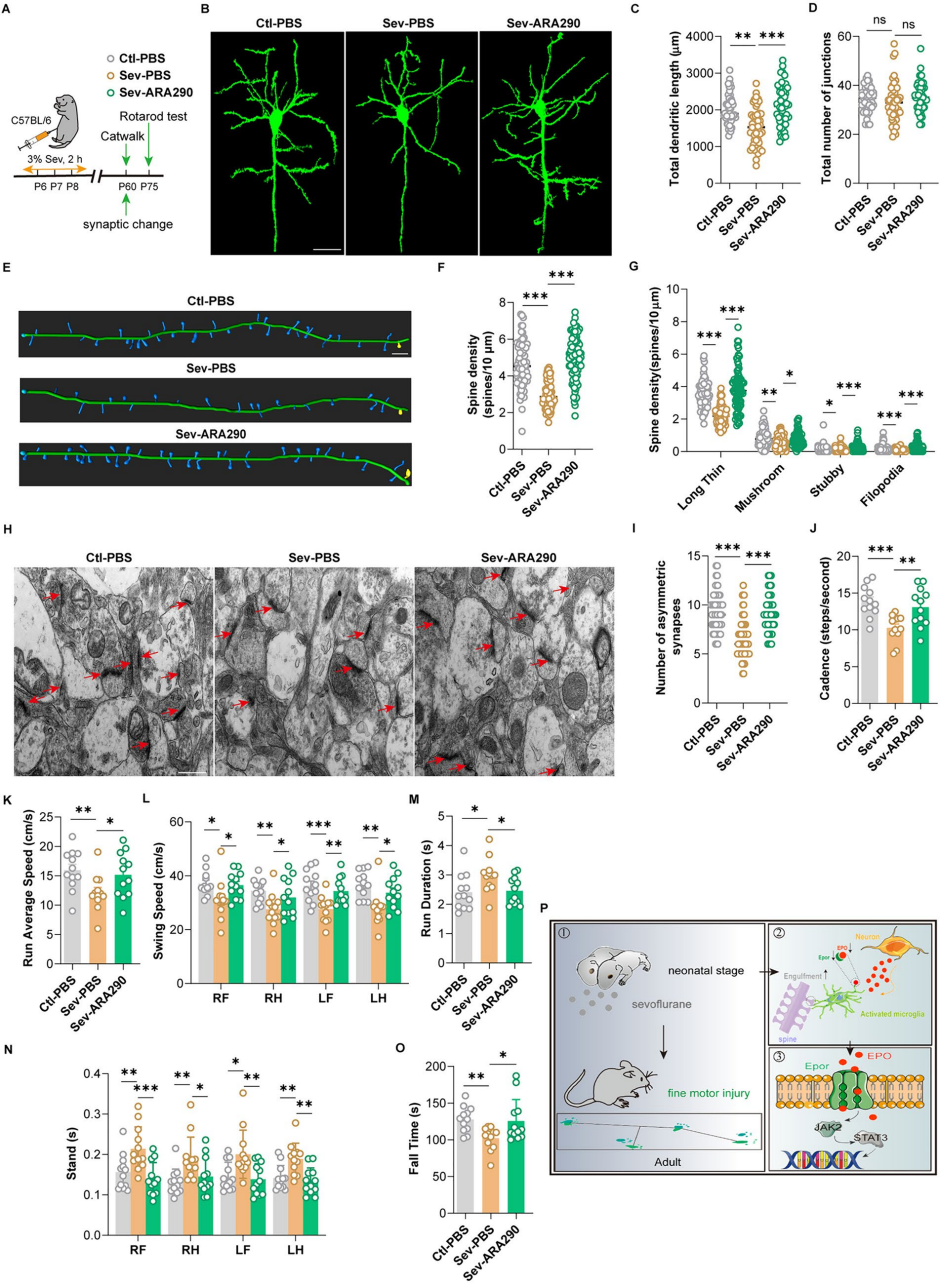
The effect of ARA290 supplementation on synapses and fine motor function of the adult mice. **A** Schematic diagram of synaptic observation and the behavioral tests. **B** Representative images of Golgi-stained pyramidal neurons in the mPFC from three groups after ARA290 supplementation (scale bar, 25 mm), *n = *6, 36 pyramidal neurons. **C**–**D** Sholl analysis of the morphology of pyramidal neurons. **E** Representative 3D reconstructed images of Golgi-stained dendrites of pyramidal neurons in the mPFC from three groups after ARA290 supplementation (scale bar, 10 mm), *n = *6, 84–90 dendrites. **F**–**G** Quantitative analysis of density of all spines (**F**) and four different types of spines (**G**) based on Golgi staining. **H** Representative images of TEM in the mPFC from three groups after ARA290 supplementation (arrows, excitatory synapse; scale bar, 0.5 mm), *n = *6. **I** Quantitative analysis of excitatory synapse. **J**–**N** Gaits analysis of three groups, *n* *=* 12. The parameters include cadence (**J**), run average speed (**K**), swing speed (**L**), run duration (**M**), and stand duration (**N**). **O** Latency of fall down of the rotarod test for three groups, *n = *12. **P** Graphical abstract: schematic diagram depicting the mechanism underlying microglial EPO/EPOR-JAK2-STAT3 signaling pathway mediated sevoflurane-induced fine motor deficits in mice. The data are denoted as the mean ± SD. *P < 0.05, **P < 0.01, ***P < 0.001, ****P < 0.0001 (Kruskal–Wallis H test with Nemenyi’s multiple-comparison test).

We performed Golgi staining to demonstrate the effect of supplementing ARA290 on long-term synaptic toxicity. The results displayed that the length of the pyramidal neurons' dendrites became longer (Fig. 6B, C), and the spinal density of pyramidal neurons was significantly increased (Fig. 6E, F) after ARA290 supplementation. The number of junctions did not decrease significantly (Fig. 6D), while the number of four kinds of dendritic spines was increased after ARA290 supplementation (Fig. 6G). The density of excitatory synapses detected by TEM returned to a level similar to the Ctl-PBS group (Fig. 6H, I). Then, we evaluated whether supplementation of ARA290 exerted a salvage effect on fine motor dysfunction. Gait analysis showed that the walking speed was significantly increased, including cadence, run average speed, and swing speed (Fig. 6J–L), and the completion and standing time were shortened after ARA290 supplementation (Fig. 6M, N). There were no remarkable differences in other static indicators, including the base of support-front/hind paws, duty cycle, max contact area, mean intensity, and run maximum variation (Fig. S1A–F). The result of the rotarod test displayed a noteworthy increase in the descending latency after supplementing ARA290 (Fig. 6O). Together, these data illustrate that supplementation of ARA290 alleviates long-lasting synaptic toxicity and fine motor dysfunction caused by repeated sevoflurane exposure in neonatal mice.

## Discussion

Several well-known clinical trials including MASK, Pediatric Anesthesia Neurodevelopment Assessment (PANDA), and General Anesthesia compared to Spinal anesthesia (GAS) studies elucidate that there is no evident relation between single short exposure of general anesthetics with aberrant neurologic behaviors [28–31]. Nevertheless, the MASK study that 997 children (411, 380, and 206 un-, singly-, and multiply-exposed, respectively) born in Olmsted County, MN from 1994 to 2007 were sampled using a propensity-guided approach and underwent neuropsychological testing at ages 8–12 or 15–20 years suggests that multiple exposures to general anesthetics is associated with decreased fine motor skills, manifested as descends in processing speed and motor coordination in subjects [8]. Numerous animal studies have also found that general anesthesia induced fine motor deficits [7, 9]. In animal research, methods for studying fine motor skills include the Beam walking test [7], MoRaG test [32], the footprint test [33], the rotarod test [34], and the Catwalk test [35]. The footprint test and Catwalk test have similar principles for gait analysis, but the latter is a new testing device with the advantages of convenient and accurate use. In this study, by using catwalk and rotarod tests, we found that the grew-up mice that were exposed to sevoflurane 3 times in their early stage of life showed fine motor deficits, namely descends in the speed of swing and cadence, as well as extensions in run duration and stand time. These findings were consistent with decreased processing speed in subjects undergoing repeated general anesthesia.

Anesthetic neurotoxicity including fine motor deficits involves diverse mechanisms and cell types, including synaptic changes, myelin sheath injury, and others. Neuroinflammation is an upstream cross-link among them [25]. Microglia are emerging as critical regulators of neuronal function and behavior in nearly every area of neuroscience, especially in neuroinflammation [36]. Recent evidence has demonstrated that pro-inflammatory circumstances triggered synapse remodeling through microglia [37–40]. Dysfunction of microglia-mediated synaptic pruning during neurodevelopment results in abnormal behaviors in adulthood such as social behaviors [41], Autism spectrum disorder (ASD) [42], and Alzheimer's disease (AD) [43]. The intact fine motor function involves complex coordination between numerous central (the premotor and motor cortex, cerebellum, basal ganglia, and corticospinal tracts) and peripheral nervous system (peripheral nerves, visuospatial, sensory, and executive function processing) structures [44]. Although the cerebellum is vital for the coordination of movement, especially in the initiation of complex pre-planned fine motor movements [45], our study found that there was no significant change in the coordination indicator in the Catwalk assay (Fig. S1A–1E). However, the mice’s processing speed, one of the major components of fine motor control [46], was significantly reduced in our study. Pre-motor and motor cortex, including mPFC, are higher cortical centers that initiate movement [47]. Multiple functional and structural imaging studies have shown that the mPFC plays a very important role in regulating the processing speed of the fine motor skills [16]. Therefore, we focus on the mPFC. Additionally, the proportion of excitatory and inhibitory neurons varies among different brain regions, with 60%–70% of excitatory neurons in the mPFC [48]. In recent years, the microglia-dependent phagocytosis of excitatory synapses has been proposed to occur in many neurological diseases [49–51]. Consistently, we found over-pruning of excitatory synapses by microglia in the mPFC in the sevoflurane-exposed mice. Namely, repeated exposure to sevoflurane resulted in microglial morphological alterations and excessive pruning of excitatory synapses by microglia in the mPFC. Whereas transient morphological alterations of microglia, the aberrant microglial function of synaptic phagocytosis was sustained until P21—the optimal period of synaptic pruning [52] in the rodent brain. In our study, Golgi staining showed that the dendritic spine density was decreased in adulthood after repeated exposure to sevoflurane. We and others have shown that both presynaptic components (including SYN and Vglut2, etc.) and postsynaptic components (including Homer and PSD95, etc.) were decreased after exposure to sevoflurane [7, 21, 53–55]. Considering some studies demonstrated that sevoflurane can increase dendritic spine density during development [56, 57], we reasoned that excessive pruning of synapses by microglia rather than reduced synaptic formation leads to behavioral changes.

Removing the redundant synapses correctly is important for the normal development and functional homeostasis of the healthy brain, which requires the collaboration of signals modulating synaptic elimination [58]. To reveal the underlying mechanism, we screened out 20 upregulated genes and 177 downregulated genes by RNA Seq (Fig. 5B–D). In the CNS, EPOR is highly expressed in microglia [17] and regulates the morphology and function of microglia to exert anti-neuroinflammatory effects through the EPO/EPOR-JAK2-STAT3 pathway [59]. Therefore, the substantial decrease in EPOR draws our attention. EPO is a hypoxia-inducible factor that is swiftly upregulated when oxygen deficiency occurs [60]. During sevoflurane anesthesia like our study, carrier gas with 60% oxygen in addition to a gas monitor was used to prevent hypoxia. In line with our findings, other literature also reported the EPO/EPOR-inhibitory effect of general anesthesia exposure in the view of anti-apoptosis [61, 62]. It's probably because most of all general anesthetics decrease the cerebral metabolic rate of oxygen. The regulation of EPO lies in hypoxia-inducible factor-1 (HIF-1), which is made up of two subunits HIF-*α* (containing HIF-1*α*, HIF-2*α,* and HIF-3*α*) and HIF-1β [63]. We detected the expression of HIF-*α* and found that it was HIF-2*α*, not HIF-1*α* (Fig. S1B–D), that was responsible for EPO suppression after repeated sevoflurane exposure. We demonstrated that inhibition of EPO/EPOR-JAK2-STAT3 signaling pathway mediated microglia activation and excessive phagocytosis of synapses after repeated sevoflurane exposure. Our results suggest a new strategy to treat such fine motor disorders by targeting the EPO/EPOR system.

For clinical use, numerous clinical trials as well as “translation-guided” studies considered recombinant human (rh) EPO or ARA290, an EPO derivative, as an efficient treatment for improvements of cognitive performances including learning, memory, attention, executive function, and processing speed [64]. Studies showed that exogenously applied ARA290 exerts neuroprotection without side effects of hematopoiesis and erythropoiesis [65]. Therefore, we selected ARA290 for the treatment of sevoflurane-induced neurotoxicity. Our data showed that at a dose of 30 μg/kg ARA290 effectively restored the activation of the EPOR-JAK2-STAT3 pathway, alleviated aberrant activation and synaptic pruning, and rescued sevoflurane-induced fine motor deficits. Thus, the cumulative evidence supports that EPOR could serve as a potential interventional target for treating sevoflurane-induced neurotoxicity in children.

The following aspects may contribute to the improvement of this study. Firstly, the hypothesis evolves that functional microglial diversity ensures the selective pruning of excitatory versus inhibitory synapses. Several studies have demonstrated that GABA-receptive microglia selectively remodel the inhibitory but not excitatory synapses [52]. An association between microglia and inhibitory synapses, as well as the roles in sevoflurane-induced fine motor deficits, needs to be investigated in further study. Secondly, since EPO is an endogenous hormone that is distributed throughout the body, the causal relationship between EPO alterations in peripheral organs and the brain should be investigated. Thirdly, the role of microglia-specific EPOR in sevoflurane-induced pathological phagocytic functions is still lack of evaluation. Transgene animals and virus targeting on microglial EPOR should be constructed for further exploration.

## Conclusions

Based on our findings, we conclude that repeated sevoflurane exposure to the newborn mice induces activation of microglia and excessive synaptic pruning by downregulating EPO/EPOR-JAK2-STAT3 signal pathway in the mPFC, which leads to fine motor deficits in the adult mice. The EPO derivative ARA290 could rescue pathological phagocytosis of microglia and subsequently alleviate sevoflurane-induced fine motor deficits. The graphical abstract is shown in Fig. 6P.

## Supplementary Information

Below is the link to the electronic supplementary material.

Supplementary file1 (PDF 1333 kb)
