# Supplementary material for: Microglial EPOR Contribute to Sevoflurane-induced Developmental Fine Motor Deficits Through Synaptic Pruning in Mice
Source: Neurosci Bull. 2024 Jun 21;40(12):1858–74. doi: 10.1007/s12264-024-01248-5 (PMC11625042; doi:10.1007/s12264-024-01248-5)
Supplement: Supplementary file 1 — Supplementary file1 (PDF 1333 kb) [file 12264_2024_1248_MOESM1_ESM.pdf]

Supplementary Information

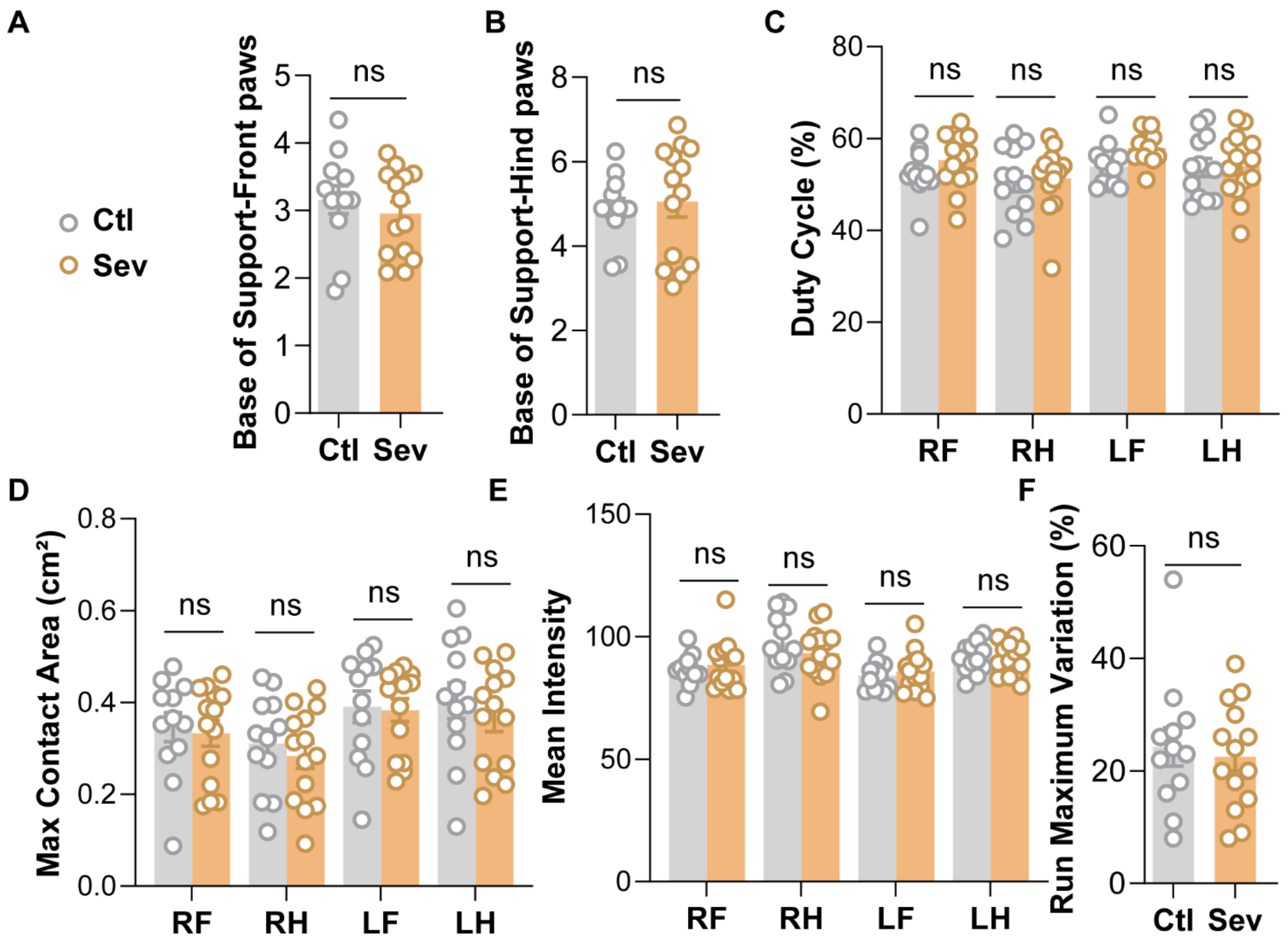

Fig. S1 There was no remarkable difference in other gait indicators of mice after repeated exposure to sevoflurane in the neonatal stage. **A**-**F**. Gaits analysis of the Ctl (*n* = 12) and Sev group (*n* = 14). The parameters include the base of support-front paws (**A**), base of support-hind paws (**B**), duty cycle (**C**), max contact area (**D**), mean intensity (**E**), and run maximum variation (**F**). The data are denoted as the mean ± SD. **P* < 0.05, ***P* < 0.01, ****P* < 0.001, *****P* < 0.0001 (unpaired separate variance estimation *t* test).

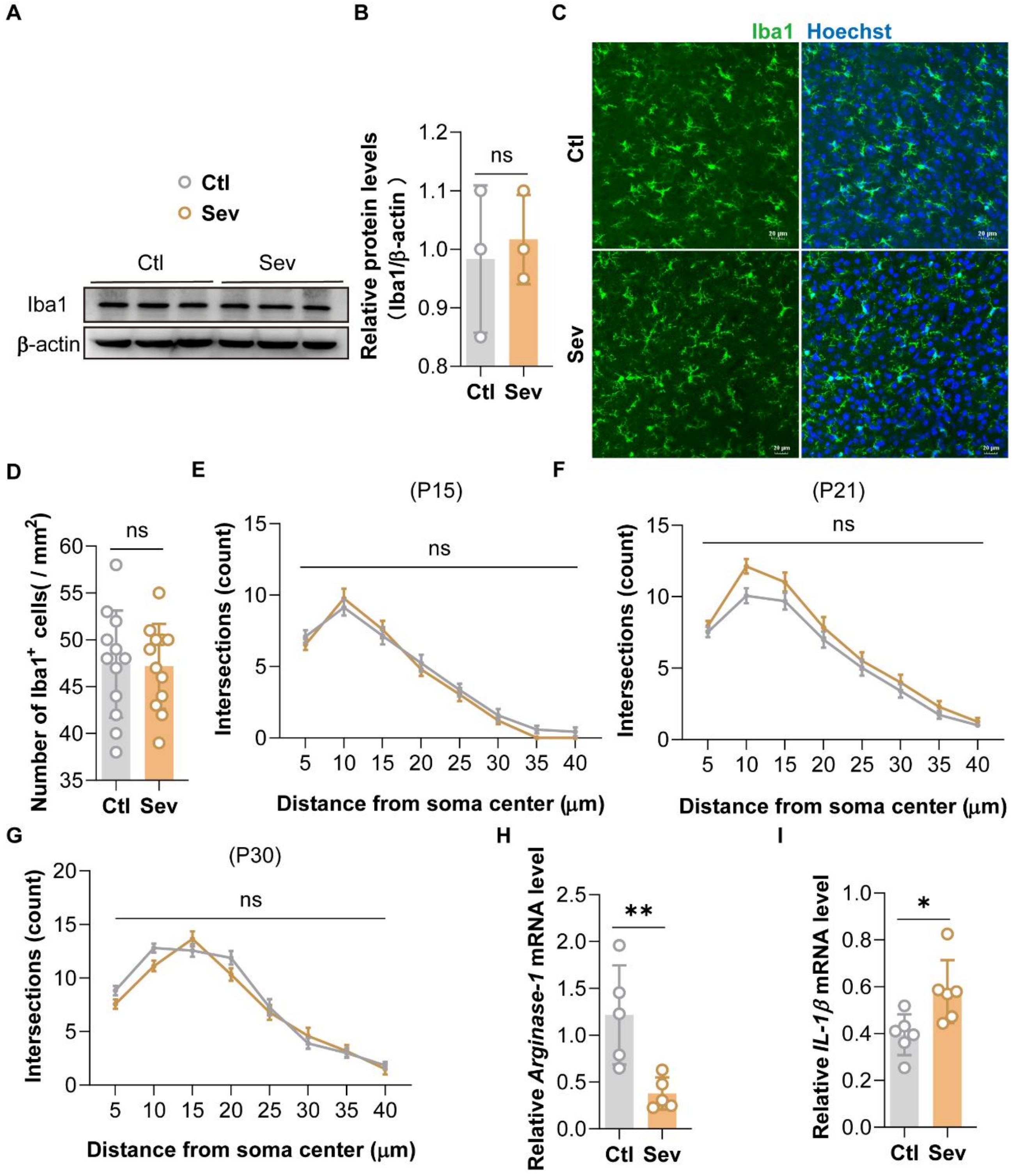

Fig. S2 The number of microglia did not change dramatically after repeated exposure to sevoflurane. **A**-**B**. The protein level (**A**) and quantification (**B**) of Iba1 in the mPFC from the Ctl and Sev group on the 3rd day after repeated exposure to sevoflurane (unpaired separate variance estimation *t*-test), n = 3. **C**. Representative images of Iba1 expression in the mPFC from the Ctl and Sev group on the 3rd

day after repeated exposure to sevoflurane (scale bar, 50 mm), $n = 6$. **D**. Quantitative analysis of Iba1+ cell number (unpaired separate variance estimation *t*-test). **E-G**. Sholl analysis of microglia morphology in the mPFC from the Ctl and Sev group on the 7th (P15) (**E**), 13th (P21) (**F**), and 22nd (P30) (**G**) days after repeated exposure to sevoflurane (Friedman's M-test). **H-I**. Quantitative mRNA expression level of Arginase-1 (**H**) and IL-1β (**I**) in the mPFC from the Ctl and Sev group on the 3rd day after repeated exposure to sevoflurane (unpaired separate variance estimation *t*-test), $n = 6$. The data are denoted as the mean ± SD. *$P < 0.05$, **$P < 0.01$, ***$P < 0.001$, ****$P < 0.0001$.

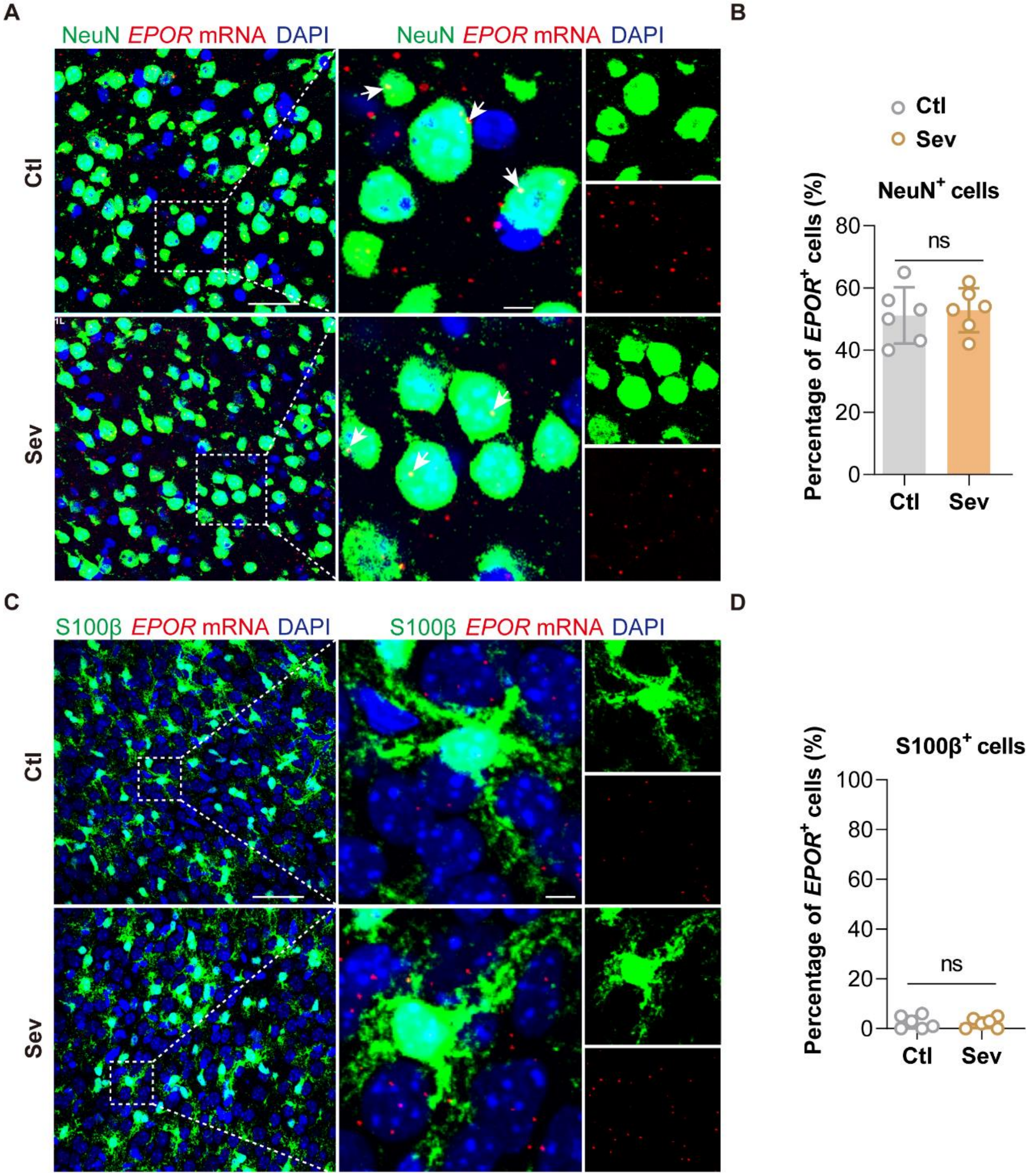

Fig. S3 EPOR expression and changes in neurons and astrocytes in the mPFC after repeated exposure to sevoflurane. **A**. RNAscope in situ hybridization and immunohistochemistry staining showing colocalization of NeuN and *EPOR* mRNA in the mPFC of the Ctl and Sev group on the 3rd day after repeated exposure to sevoflurane, scale bars, left: 25 mm, middle and right: 10 mm. **B**. Quantification

of the percentage of *EPOR*$^+$ cells in NeuN$^+$ cells, n = 6. **C**. RNAscope in situ hybridization and immunohistochemistry staining showing colocalization of S100β and *EPOR* mRNA in the mPFC of the Ctl and Sev group on the 3rd day after repeated exposure to sevoflurane, scale bars, left: 25 mm, middle and right: 10 mm. **D**. Quantification of the percentage of *EPOR*$^+$ cells in S100β$^+$ cells, n = 6. The data are denoted as the mean ± SD. *P < 0.05, **P < 0.01, ***P < 0.001, ****P < 0.0001 (unpaired separate variance estimation t test).

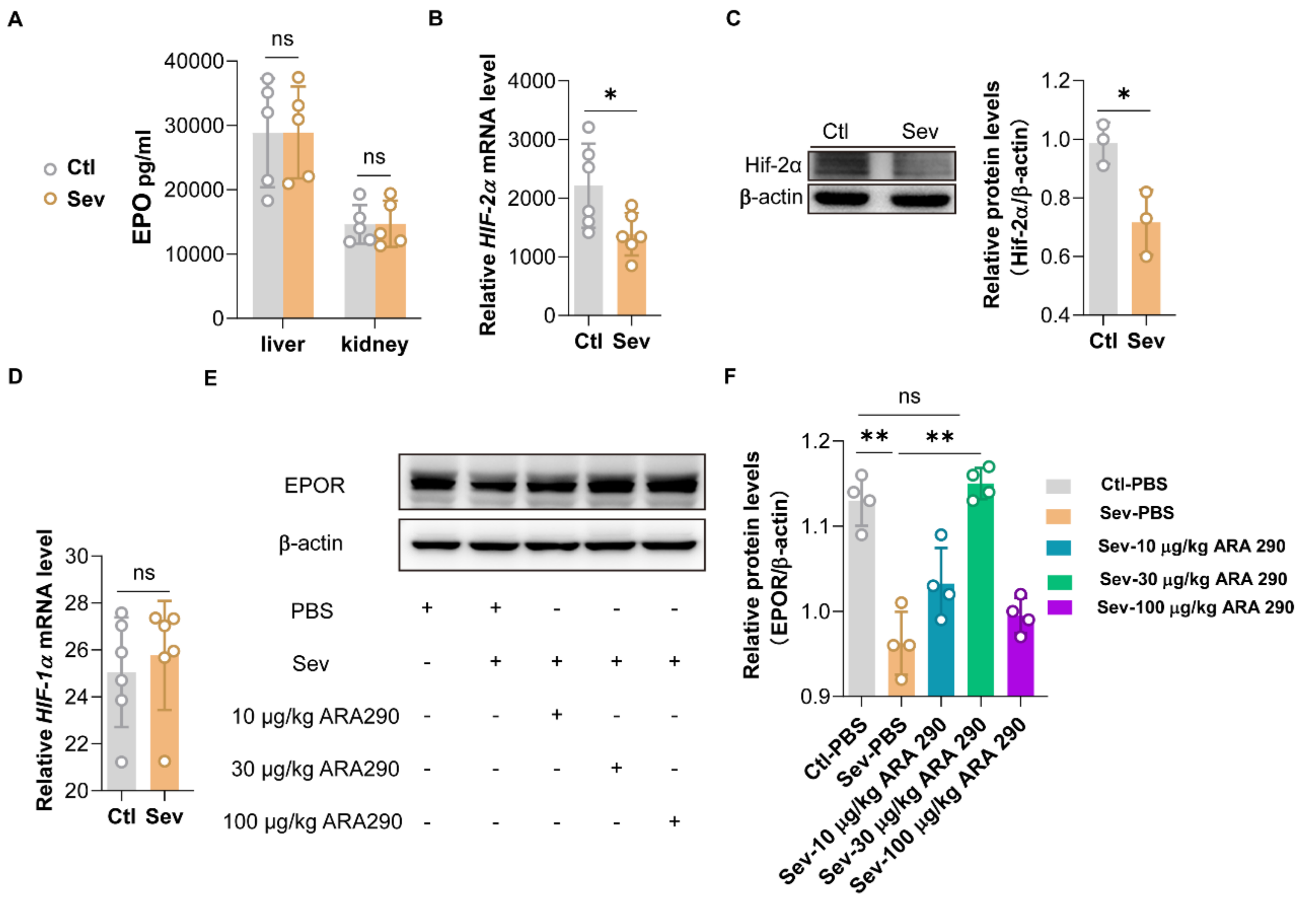

Fig. S4 The expression of HIF-2α, rather than HIF-1α, was decreased in the mPFC after repeated exposure to sevoflurane. **A**. The expression level of EPO in the liver and kidneys of mice between the Ctl and Sev group (unpaired separate variance estimation *t*-test), n = 5. **B** and **D**. Quantitative mRNA expression level of HIF-2α (**B**) and HIF-1α (**D**) in the mPFC from the Ctl and Sev group on the 3rd day after repeated exposure to sevoflurane (unpaired separate variance estimation *t*-test), n = 6. **C**. The protein level and quantification of HIF-2α in the mPFC from the Ctl and Sev group on the 3rd day after repeated exposure to sevoflurane (unpaired separate variance estimation *t*-test), n = 3. **E**. Expression of EPOR in the mPFC under different concentrations of ARA290. **F**. Quantification of EPOR protein levels (Kruskal-Wallis H test with Nemenyi's multiple-comparison test), n = 4. The data are denoted as the mean ± SD. *$P < 0.05$, **$P < 0.01$, ***$P < 0.001$, ****$P < 0.0001$.

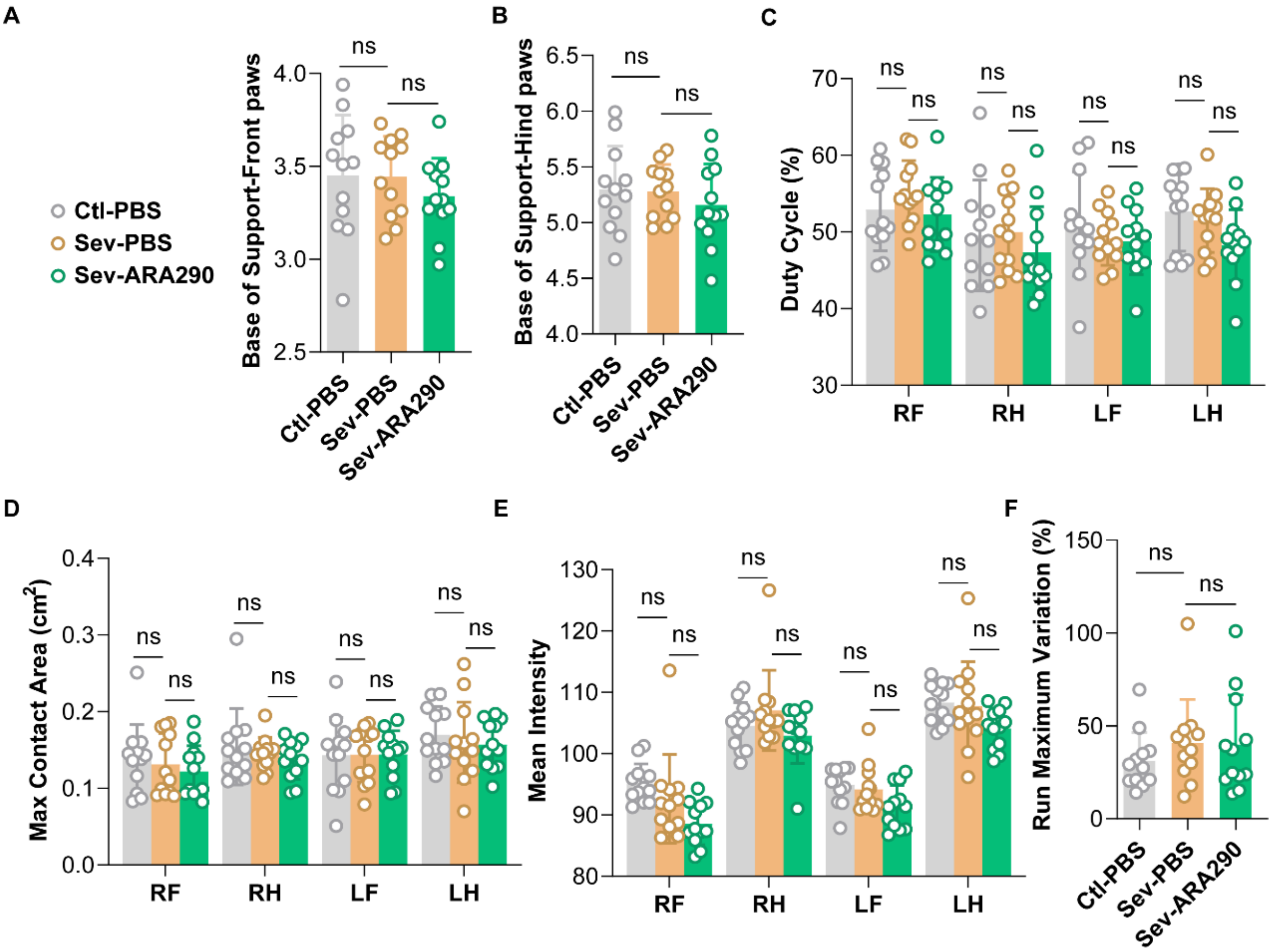

Fig. S5 There was no remarkable difference in other gait indicators of mice after supplementing ARA290. **A**-**F**. Gaits analysis of three groups, $n = 12$. The parameters include the base of support-front paws (**A**), base of support-hind paws (**B**), duty cycle (**C**), max contact area (**D**), mean intensity (**E**), and run maximum variation (**F**). The data are denoted as the mean ± SD. *$P < 0.05$, **$P < 0.01$, ***$P < 0.001$, ****$P < 0.0001$ (Kruskal-Wallis H test with Nemenyi's multiple-comparison test).

Table S1 The primers used in this study

Gene Name	Sense (5′-3′)	Anti-sense (5′-3′)
GADPH	GGTGAAGGTCGGTGTGAACG	CTCGCTCCTGGAAGATGGTG
EPOR	GGGCTCCGAAGAACTTCTGTG	ATGACTTTCGTGACTCACCCT
IL-1β	GCAACTGTTCCTGAACTCAACT	ATCTTTTGGGGTCCGTCAACT
Arginase-1	CTCCAAGCCAAAGTCCTTAGAG	AGGAGCTGTCATTAGGGACATC
EPO	ACTCTCCTTGCTACTGATTCCT	ATCGTGACATTTTCTGCCTCC
HIF-1*α*	ACCTTCATCGGAAACTCCAAAG	CTGTTAGGCTGGGAAAAGTTAGG
HIF-2*α*	CTGAGGAAGGAGAAATCCCGT	TGTGTCCGAAGGAAGCTGATG
